# Osteogenic Properties of PBLG-g-HA/PLLA Nanocomposites

**DOI:** 10.1371/journal.pone.0105876

**Published:** 2014-09-03

**Authors:** Lan Liao, Shuang Yang, Richard J. Miron, Junchao Wei, Yufeng Zhang, Meng Zhang

**Affiliations:** 1 School of Materials Science and Engineering, Nanchang University, Nanchang, PR China; 2 School of Stomatology, Nanchang University, Nanchang, PR China; 3 The State Key Laboratory Breeding Base of Basic Science of Stomatology (Hubei-MOST) & Key Laboratory of Oral Biomedicine Ministry of Education, School & Hospital of Stomatology, Wuhan University, Wuhan, PR China; 4 Department of Oral Implantology, School of Stomatology, Wuhan University, Wuhan, PR China; Universita’ degli Studi del Salento, Italy

## Abstract

New development of biomaterial scaffolds remains a prominent issue for the regeneration of lost or fractured bone. Of these scaffolds, a number of bioactive polymers have been synthesized and fabricated for diverse biological roles. Although recent evidence has demonstrated that composite scaffolds such as HA/PLLA have improved properties when compared to either HA or PLLA alone, recent investigations have demonstrated that the phase compatibility between HA and PLLA layers is weak preventing optimal enhancement of the mechanical properties and making the composites prone to breakdown. In the present study, poly (γ-benzyl-L-glutamate) modified hydroxyapatite/(poly (L-lactic acid)) (PBLG-g-HA/PLLA) composite scaffolds were fabricated with improved phase compatibility and tested for their osteogenic properties in 18 Wistar female rats by analyzing new bone formation in 3 mm bilateral femur defects in vivo. At time points, 2, 4 and 8 weeks post surgery, bone formation was evaluated by µ-CT and histological analysis by comparing 4 treatment groups; 1) blank defect, 2) PLLA, 3) HA/PLLA and 4) PBLG-g-HA/PLLA scaffolds. The in vivo analysis demonstrated that new bone formation was much more prominent in HA/PLLA and PBLG-g-HA/PLLA groups as depicted by µ-CT, H&E staining and immunohistochemistry for collagen I. TRAP staining was also utilized to determine the influence of osteoclast cell number and staining intensity to the various scaffolds. No significant differences in either staining intensity or osteoclast numbers between all treatment modalities was observed, however blank defects did contain a higher number of osteoclast-like cells. The results from the present study illustrate the potential of PBLG-g-HA/PLLA scaffolds for bone tissue engineering applications by demonstrating favorable osteogenic properties.

## Introduction

Bone fracture and damage are important health concerns worldwide. Although bone is known to have excellent self-healing properties when compared to other body tissues [1], patients suffering from large sized defects often require bone substitute materials in order to promote new bone formation and fill large bone voids [2]. Typically bone substitutes necessitate a number of physical and mechanical properties making them suitable for clinical application including biocompatibility, safety, mechanical stability, advantageous surgical handling, and an osteoconductive surface which supports cell and tissue ingrowth [3].

Hydroxyapatite (HA) is a commonly used bone replacement material due to its similar chemical composition with the inorganic phase of bone, making it one of the most popular choices for clinicians working in the field of bone repair and bone tissue engineering [4]–[6]. Despite its widespread use, the main drawback of HA are its inherent brittleness and poor mechanical properties as a replacement material during load-bearing bone repair [7], . Poly (L-lactic acid) (PLLA) is a biomaterial with an excellent combination of biocompatibility, bio-degradability and mechanical properties [9]–[11]. However, a number of drawbacks such as limited osteoconduction and poor cell–material interactions have restricted their application for bone tissue healing [12].

To overcome the drawbacks of these materials, it was recently demonstrated that the coating of PLLA nanofibers with HA particles improved the biological properties of PLLA alone by improving osteoblast differentiation and ectopic bone formation in animal models [12], [13]. Although the HA/PLLA nanocomposites have improved properties when compared to either HA or PLLA alone, recent investigations have demonstrated that the phase compatibility between HA and PLLA layers are weak, the HA particles aggregate heavily in the PLLA matrix, preventing optimal enhancement of the mechanical properties and making the composites prone to breakdown [14], [15].

In order to prevent the aggregation of HA nanoparticles and tune the phase compatibility between HA and polymers, various methods have been used to graft polymers on the surface of HA. Previously, Chen’s group have prepared polypeptide poly (γ-benzyl-L-glutamate) modified HA nanoparticles (PBLG-g-HA) by the ring opening polymerization of γ-benzyl-L-glutamate N-carboxyanhydride (BLG-NCA), and their research has demonstrated that PBLG-g-HA can maintain higher colloidal stability and improve cell adhesion when compared to that of pure HA. In addition, the mechanical properties of PBLG-g-HA/PLLA composites were higher than that of HA/PLLA [8], [16]. The aim of the present study was to extrapolate the findings from recent studies eliciting the advantages of PLLA incorporating with PBLG-g-HA nanoparticles and create a homogeneous scaffold of PBLG-g-HA/PLLA. These scaffolds were then compared for their ability to form new bone formation in a rat femur defect model when compared to blank, PLLA and HA/PLLA scaffolds.

## Materials and Methods

### 1. Preparation and characterization of the PBLG–g-HA/PLLA

PBLG-g-HA was prepared according to previously published work [16]. Briefly, HA nanoparticles were firstly treated with 3-aminopropyl triethoxy silane, connecting amino groups on the surface of HA, and then PBLG-g-HA was acquired via the ring opening polymerization of BLG-NCA with amino group functionalized HA as initiators. The weight loss of PBLG-g-HA was determined by thermogravitational analysis (TGA) at 21.1%.

PBLG-g-HA/PLLA porous scaffolds were prepared by the thermal induced phase separation method. Firstly, 0.1 g PBLG-g-HA was dispersed in 10 mL dioxane under ultrasonic, and then 0.9 g PLLA was added into the suspension system under vigorous stirring. When PLLA was completely dissolved, the mixture was frozen in liquid nitrogen, in the end the freezed dioxane was removed by lyophilization, and the 3D porous scaffolds were obtained. PLLA and HA/PLLA scaffolds were also prepared with the same method. Morphology of the scaffolds was observed with a scanning electronic microscopy (SEM, FEI Quanta 200 SEM).

### 2. The in vivo osteogenic properties of composite scaffolds

The bone forming ability of each scaffold was evaluated in Wistar female rats with defects created in their femoral condyles by surgery. At time points, 2, 4 and 8 weeks post surgery, bone formation was evaluated by µ-CT and histological analysis.

#### 2.1. Animals and surgical protocols

Animal handling and surgical procedures were under the direction of the guidelines for animal care and use committee of Wuhan University, People’s Republic of China, and approved by the Ethics Committee at the School of Dentistry, Wuhan University, China. Before the experiment, all the animals (2-month-old mature female Wistar rats were used for this study) were kept in the vivarium of the Stomatology of Wuhan University and supported with food and water ad libium. The operations were performed in germ-free conditions with a tender surgical technique.

#### 2.2. Femur defect model

Prior to the start of surgery, 18 wistar female rats were divided into 3 groups randomly corresponding to the 3 treatment periods. At each time point, 1 control+3 scaffolds were considered as follows: 1) control empty group, 2) PLLA, 3) HA/PLLA and 4) PBLG-g-HA/PLLA. At each time point, 4 defects were filled with each of the 3 scaffolds or left blank giving a total of 12 defects treated with each scaffold at each time point. Each animal received 2 treatment groups (one in each femur) for a total of 6 animals per time point.

Femur defect drilling was carried out under general anaesthesia by intraperitoneal injection of 10% chloraldurat (4 mL/kg body weight). Then a linear skin incision of approximately 1 cm in the distal femoral epiphysis was made and the muscles were dissected bluntly to expose the femoral condyle. Following this, a 3-mm-diameter anteroposterior bicortical channel was created perpendicular to the shaft axis by removing cancellous trabecular bone using a 2.8 mm pioneer drill at a speed of 1850 rotations/min as previously described [17]–[19]. The contact surface between drill and bone tissues was kept cool with a 0.9% physiological saline to avoid injury to the surrounding tissues. After flushing the hole with a 0.9% physiological saline to remove bone fragments from the cavity, the scaffolds were cut into 2×2×5 mm pieces to fit the hole and then the pieces were gently placed to fill the defects according to group allocation. At last the muscle and skin above the defect were closed respectively, and a single intramuscular dose of gentamycin 40,000 IU/mL was administered to prevent the postoperative infection.

Rats in different groups were sacrificed by cervical dislocation respectively at 2, 4 and 8 weeks after the surgery. All femurs were removed and collected for the follow-up micro-computerized tomography (µ-CT) analysis and histological studies.

#### 2.3. Micro-CT analysis of bone regeneration in the femoral defect

A µ-CT imaging system (µ-CT50, Scanco Medical, Basersdorf, Switzerland) was used in this experiment as previously described [20]. All the samples were fixed in 4% formaldehyde for 12 h at room temperature and then flushed with water for 3 h. Following these, the samples were scanned for the bone regeneration in the defect by µ-CT at a global thresholding procedure with a value approximating 1.20 g/cm^3^ to distinguish mineralized tissue from non-mineralization tissue. Scanning was performed at 55 kV and 114 µA with a thickness of 0.048 mm per slice in medium-resolution mode, 1024 reconstruction matrix, and 200 ms integration time. After 3D reconstruction, the bone volume faction (BV/TV) were automatically determined for identification of all the samples, while bone volume fraction in defect regions were used to evaluate new bone formation, using a protocol provided by the manufacturer of the micro-CT scanner.

#### 2.4. Histological study

Following the µ-CT scan, samples were decalcified in 10% Ethylenediaminetetraacetic acid (EDTA) which was changed every 3 days for 3 weeks, and then dehydrated in a series of graded concentration of alcohol from 70% to 100%. Following dehydration, samples were de-alcoholised in n-butanol for one night before being embedded in paraffin.

Following embedding, samples were cut into 5 mm thick blocks and the slices were mounted on polylysine-coated microscope slides as previously described [20]. Hematoxylin and eosin (H&E) staining, Safranin O staining (Sigma #S2255; SigmaeAldrich, St. Louis, USA.) and tartrate-resistant acid phosphatase (TRAP) staining (Sigma #387A; SigmaeAldrich, St. Louis, USA) were used according to manufacturer’s protocol for general histological studies.

At the same time, the expression of type I collagen (COL I) was evaluated by immunohistochemical assessment. Sections were deparaffinised in xylene two times for 10 minutes, followed by soaking in a series of graded alcohol from 100% to 70% which was changed every two minutes, and washed with phosphate buffered solution (PBS) for 5 minutes. Following washing, sections were incubated with hydrogen peroxide for 15 minutes at room temperature, washed with PBS three times for 3 minutes each, incubated with bovine serum albumin (BSA) for 15 minutes at 37°C, then the sections were incubated with the primary antibody for COL I (1∶100) (Boster SA2005, Boster Co., China) for 2 h at 37°C. After washing 3 times with PBS they were incubated with their secondary antibody for 20 minutes and made visible after the addition of Diaminobenzidine (DAB). Sections were counterstained with hematoxylin for 45 s, flushed with running water for 2 minutes, and dehydrated with an ascendant series of ethanol solutions, cleared with xylene, and then mounted with coverslips. All the sections were observed by light microscopy as previously described [20].

#### 2.5 Statistical analysis

All data analysis was performed using SPSS 19.0 software. Means and standard deviation (SD) were calculated and the statistical significance of differences among each group examined by one-way ANOVA and SNK-t test. The significance was set at P<0.05.

## Results

### 1 Characterization of PBLG-g-HA/PLLA nanocomposites

**Figure 1:**
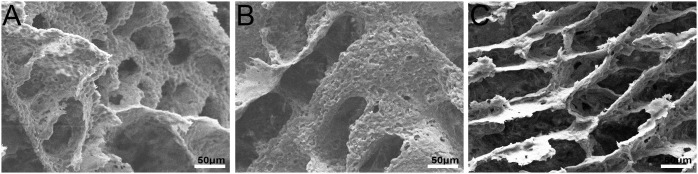
SEM images for porous scaffolds of PLLA (A), HA/PLLA (B), PBLG-g-HA/PLLA (C). (bar = 50 µm).

Porous scaffolds of PLLA, HA/PLLA and PBLG-g-HA/PLLA were prepared by the thermal induced phase separation method. The SEM images (Figure 1 A, B and C) showed that porous structures were successfully formed. The SEM images of PLLA scaffolds demonstrated a porous microsctructure (Fig. 1A) that when modified with HA was able to increase the micro-porosities of the material surface (Fig. 1B). The PBLG-g-HA/PLLA scaffolds (Fig. 1C) also demonstrated both macro and micro-porosities consistent with that of the HA/PLLA scaffolds.

### 2 µ-CT analysis of bone regeneration in the femoral defect

**Figure 2:**
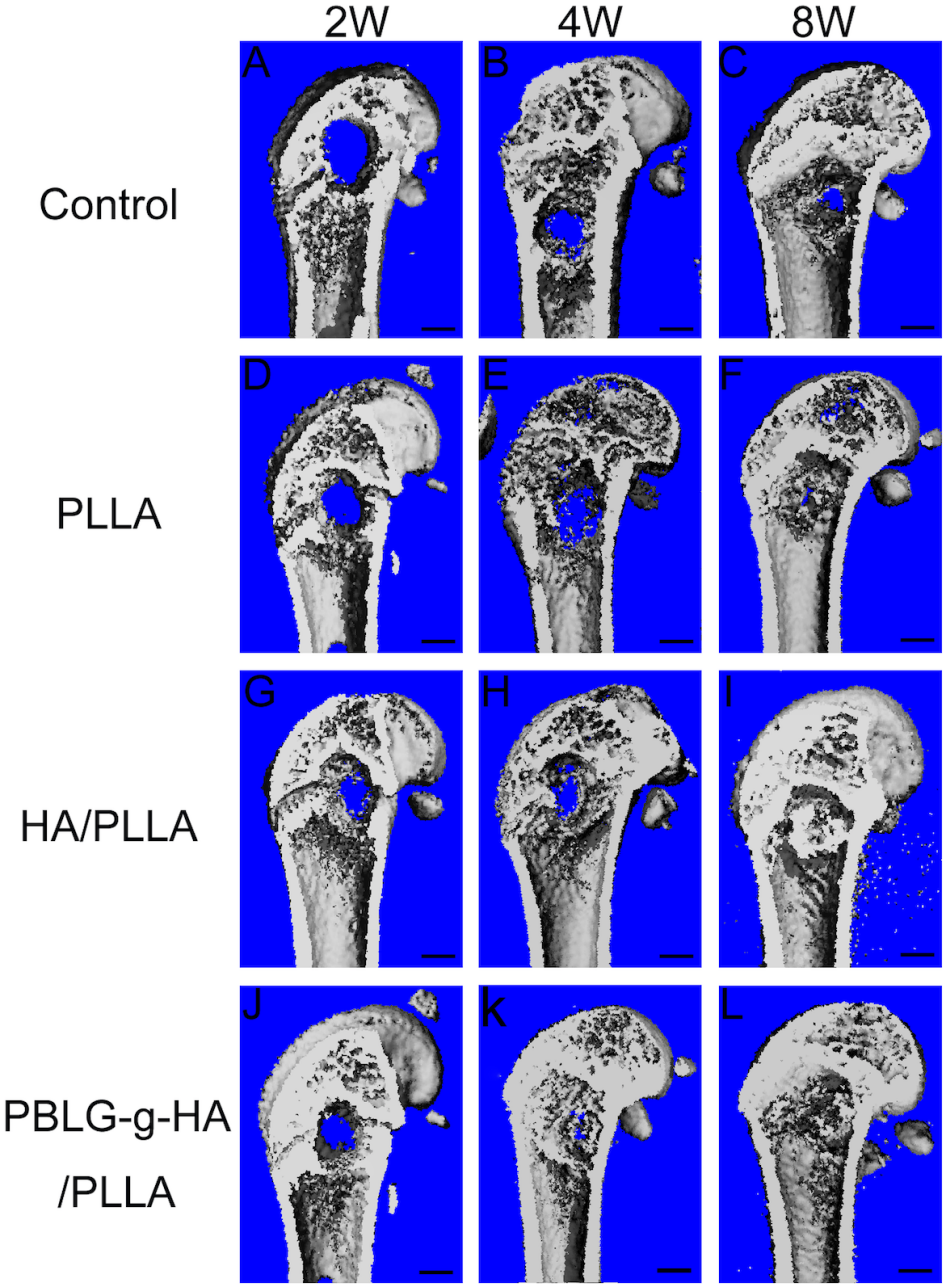
In vivo bone formation assessed by µ- CT of control (A–C), PLLA (D–F), HA/PLLA (G–I) and PBLG-g-HA/PLLA (J–L) scaffolds at 2, 4 and 8 weeks post implantation. (n = 6 animals per time point).

**Figure 3:**
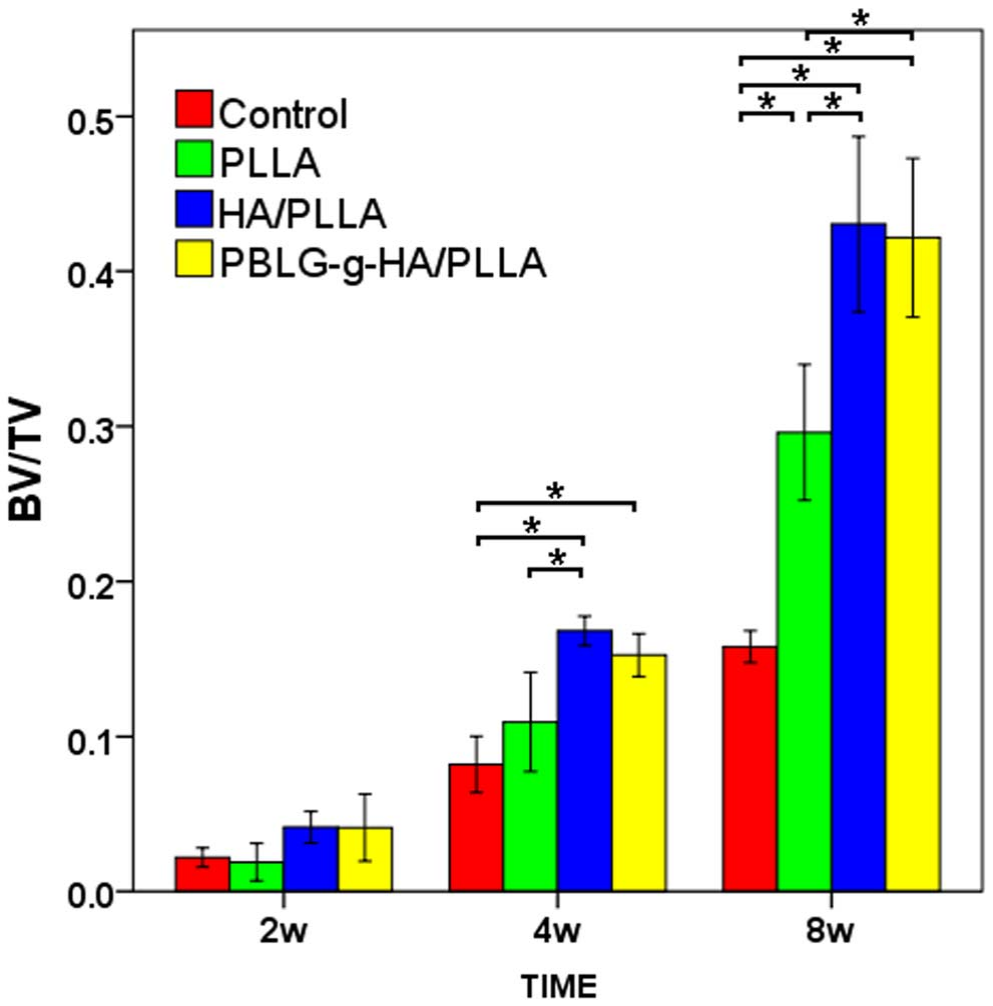
Quantification of the mineralized areas as depicted by new bone volume/total volume (BV/TV). (n = 6 animals per time point, *p<0.05).

The images of bone formation for each group were performed by 3D reconstruction shown in figure 2. The result demonstrated that all three biomaterials had some form osteogenic ability by demonstrating gradual defect fill from 2 to 8 weeks when compared to control blank defects. Quantitative analysis revealed that the bone volume (BV/TV) in the PLLA/HA and PBLG-g-HA/PLLA were superior to that of PLLA group alone (Fig. 3). No significant difference between PLLA/HA and PBLG-g-HA/PLLA was observed for their ability to form new bone formation from 2 to 8 weeks post implantation (Fig. 3).

### 3 Histological observation and immunohistochemical assessment

**Figure 4:**
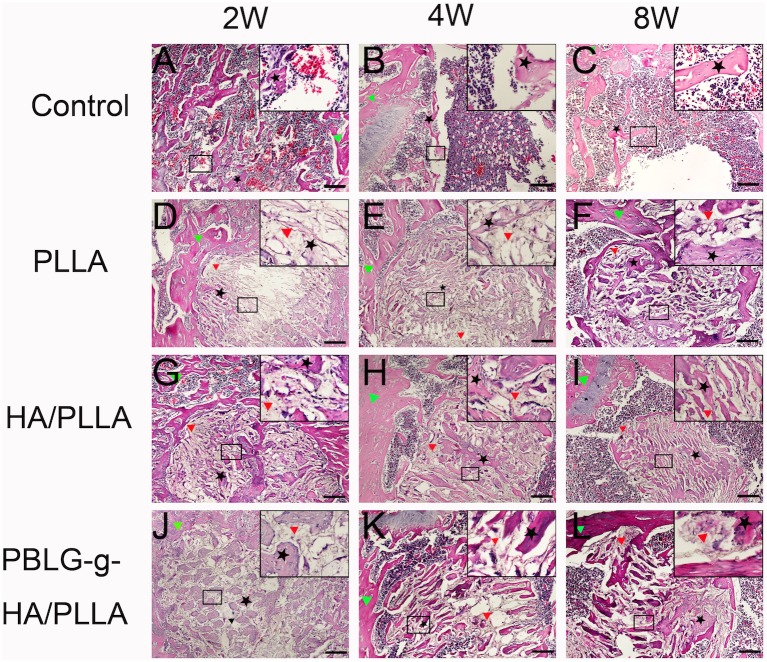
Representative HE staining of bone formation within defects 2, 4 and 8 weeks post-surgery. Abundant new bone formation was found in HA/PLLA (G, H, I) and PBLG-g-HA/PLLA (J, K, L) scaffolds, moderate bone deposition was found in pure PLLA scaffolds (D, E, F), and only minor new bone were found at the borders of drill controls (A, B, C). Black pentagon represented new bone, red triangle represented scafffolds, while the green triangle represented native bone (bar = 200 µm).

**Figure 5:**
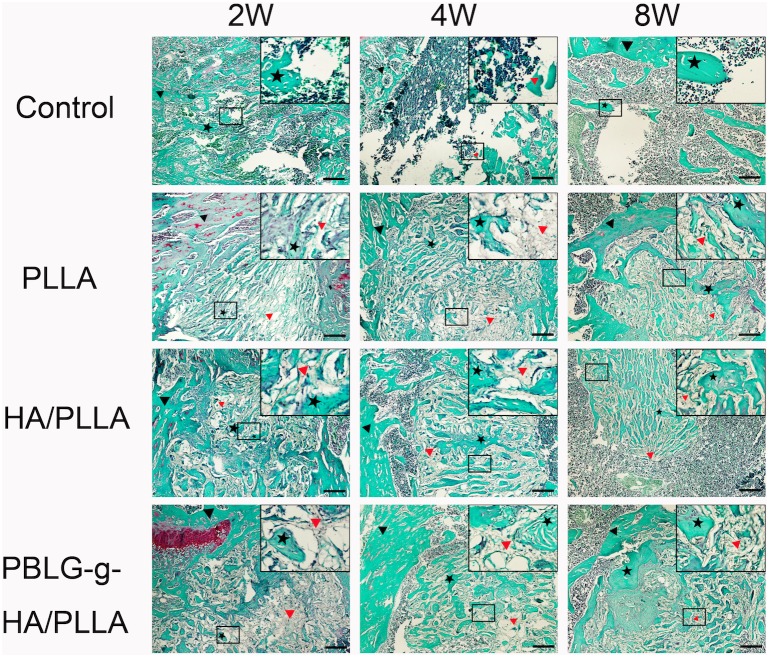
Representative Saffarin O staining of bone formation within defects 2, 4 and 8 weeks post-surgery. Abundant new bone were found in HA/PLLA (G, H, I) and PBLG-g-HA/PLLA (J, K, L) scaffolds, moderate bone deposition was found in pure PLLA scaffolds (D, E, F),and only minor new bone formation found in the drill controls (A, B, C). Black pentagons represented new bone, red triangle represented scaffolds, while black triangles represented native bone (bar = 200 µm).

H&E (Fig. 4) and Safranin O (Fig. 5) staining also confirmed abundant new bone formation in defects filled with HA/PLLA and PBLG-g-HA/PLLA scaffolds when compared to control and pure PLLA scaffolds. H&E staining at 2 weeks post-implantation revealed new tiny capillaries in the defect area of the control group as well as signs of inflammatory corpuscle and adipose tissue (Fig. 4A). In the defects receiving scaffolds, newly formed bone began to form on the scaffold surface as well as surrounding the border defects with the highest levels found around the PBLG-g-HA/PLLA at 2 weeks (Fig. 4A, D, G, J). At 4 weeks post implantation, bone remodeling became apparent bone became evident in all 3 defects filled with biomaterials (Fig. 4E, H, K). In the control group, the defect area remained notably filled with blood vessels, inflammatory corpuscle and adipose tissue and only minor new bone formation was observed on the border of the defects (Fig. 4B). At 8 weeks, osteocytes were observed in the mineral bone matrix in defects filled with HA/PLLA and PBLG-g-HA/PLLA scaffolds and the defects showed signs of fully enclosing the created defects (Fig. 4). In the control defects, a large area remained unfilled in the center of the created defects (Fig. 4C). Safranin O staining was used to observe endochondral bone formation (Fig. 5). From the panel disply in Figure 5, we can clearly note the ability for both HA/PLLA and PBLG-g-HA/PLLA scaffolds to enhance bone formation via endochondral formation (Fig. 5).

**Figure 6:**
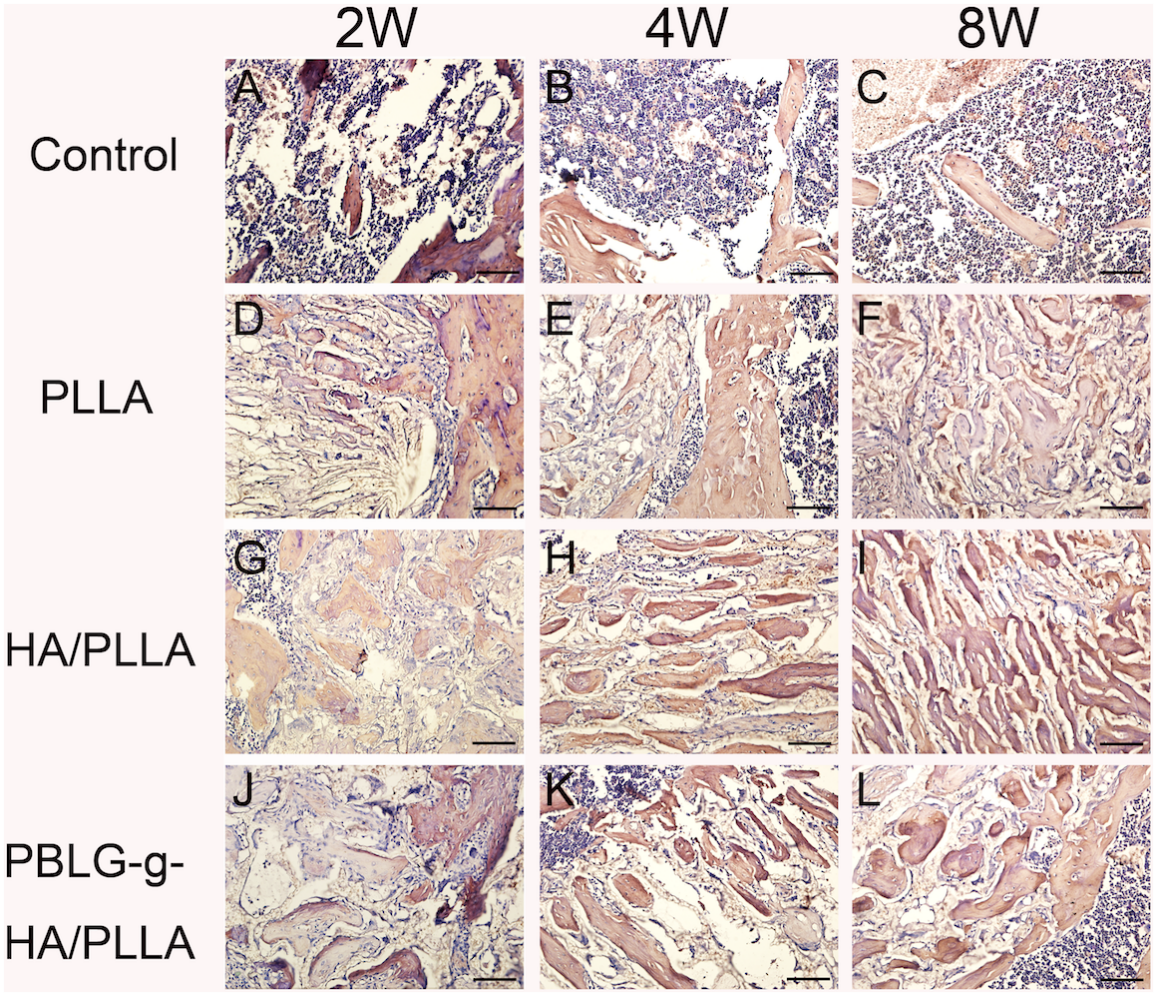
Representative immunohistological detection of COL I of bone formation within defects 2, 4 and 8 weeks post-surgery. The COL I was positively expressed at 2, 4 and 8 weeks in PLLA, HA/PLLA and PBLG-g-HA/PLLA scaffold. The result suggested that the HA/PLLA group had the greatest staining intensity (bar = 100 µm).

**Figure 7:**
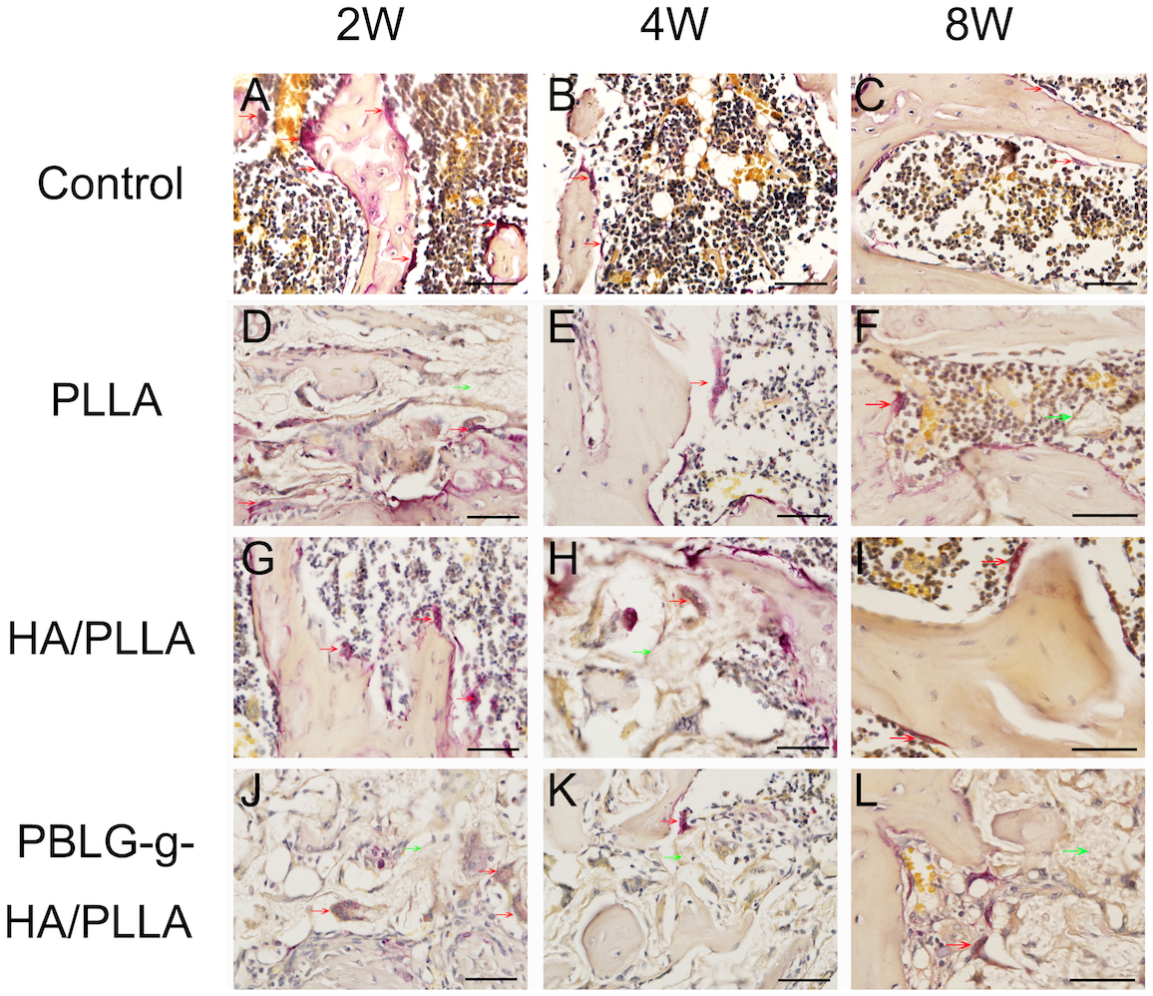
Representative TRAP staining of bone formation within defects 2, 4 and 8 weeks post-surgery. TRAP positive cells (osteoclasts) were found in all the four groups. The red arrow means osteoclast, the green arrow means the rest biomaterial. (bar = 50 µm).

**Figure 8:**
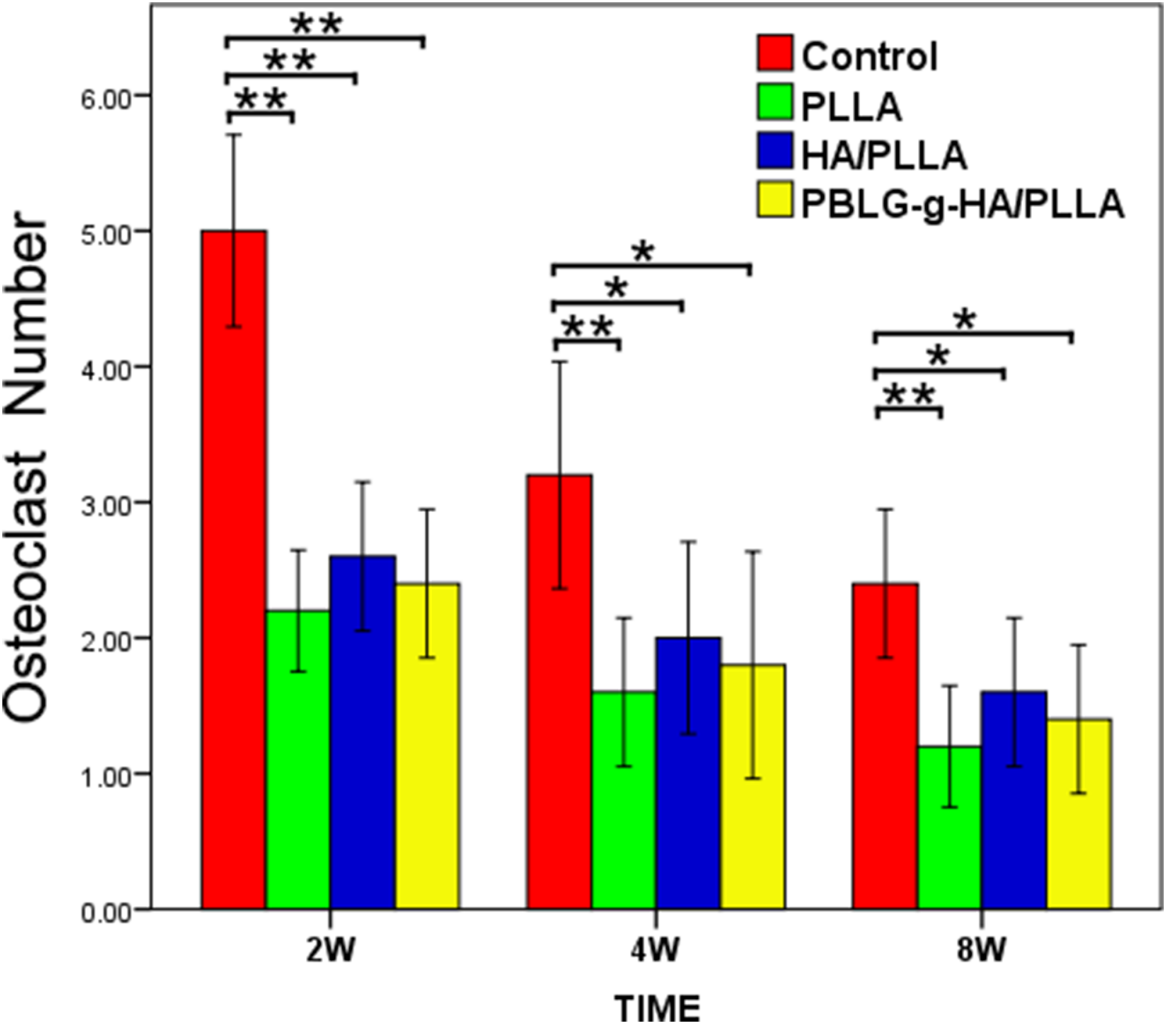
Osteoclast number in defect sites at 2, 4 and 8 weeks post implantation. (*p<0.05, **p<0.01).

COL I immunohistochemical analysis further demonstrated an increase in bone matrix deposition and maturation of bone formation sites in the defects receiving scaffolds (Fig. 6). Interestingly, the result suggest that the HA/PLLA and PBLG-g-HA/PLLA scaffolds demonstrated the highest levels of staining intensity when compared to all other treatment options (Fig. 6). To investigate the bone remodeling process, TRAP staining was conducted at 2, 4 and 8 weeks to describe the osteoclastic resorption among the four treatment options (Fig. 7). It was observed that TRAP staining did not vary much between treatment groups and a count of the number of osteoclasts that were located within the defect margins did not produce any significantly statistic differences between treatment groups (Fig. 8). However, blank defects contained a higher number of osteoclast-like cells at all time points when compared to all other treatment groups (Fig. 8).

## Discussion

The purpose of the present study was to study the osteogenic ability of new nanocomposite scaffolds PBLG-g-HA/PLLA. Many studies have previously demonstrated that the addition of HA to bone scaffolds is able to improve the osteoconductivity, biocompatibility and similarity to bone mineral in composition, size, and morphological structure. However, its apparent brittleness limits its application in load-bearing bone repair [21]–[23]. For these reasons biopolymers like PLLA, which are fabricated from renewable agriculture materials and then biodegraded back to water and carbon dioxide, have been used widely as tissue engineering scaffolds [24]. In the present study, we combined both HA and PLLA to overcome the shortcomings of each material and unite their advantages to achieve better mechanical and biomaterial properties [8]. At present, the use of HA/PLLA scaffolds [25] has demonstrated good material degradation profiles in tissues, biocompatibility as well as mechanical and crystallization properties [26], [27]. Where the material has drawbacks are in the phase compatibility between HA and PLLA which has previously been described as poor and has contributed to possible material failure [4]. To overcome this shortcoming, the goal of the present study was to fabricate a modified HA (PBLG-g-HA) to obtain PBLG-g-HA/PLLA scaffolds by thermal induced phase separation method.

In the present study, PBLG brushes were modified in order to strengthen the compatibility between HA and PLLA composites. PBLG-g-HA was synthesized according to our previous work [28] where the structure, crystalline, dispersion test and biocompatibility were ideal for a bone grafting material. The polymer brushes (PBLG) are used to improve the phase compatibility between HA and PLLA, and thus have positive effect on its mechanical properties [29]. Furthermore, the PBLG brushes may also affect thermal properties of the composites [30].

These newly developed scaffolds with modified BMLG brushes were recently characterized for their material properties (Liao et al. 2014, accepted for publication). The purpose of the present study was to compare their osteogenic ability in an animal model by implanting the scaffolds into bone defects in rat femurs and analyse their bone forming abilities. As observed in the micro-CT data, only minor bone healing was observed in the defects treated with PLLA scaffolds at the borders of the treated defects. In contrast, significantly new bone formation was observed in PBLG-g-HA/PLLA scaffolds and HA/PLLA scaffolds. It has previously been demonstrated that one of the main advantages of incorporating HA into PLLA scaffolds is to increase the 3-dimensional microporosity [26], [31]. The advantages of a 3-D porous structure has previously been demonstrated to improve cell growth and differentiation by improving cell attachment and proliferation from within the scaffold [32], [33].

The analysis of osteoclastogenesis was performed by TRAP staining, a well-known marker for osteoclast differentiation [34], [35]. The results from the present study did not seem to influence the ability for each scaffold to affect either TRAP staining intensity or osteoclast number. Only very little TRAP positive cells were found surrounding the scaffolds with little to no activity. Future research with longer time periods may be necessary to fully investigate the effect of these various scaffolds on the bone remodeling process at later time points once the material degradation is underway.

In conclusion, we demonstrate within the present study a new type of porous scaffold composed of PBLG-g-HA/PLLA prepared for bone tissue healing. The in vivo bone repair experiments demonstrated that the PBLG-g-HA/PLLA scaffolds induced higher levels of new bone formation and showed little effect on osteoclastogenesis. The results from the present study also confirm the additional use of HA to PLLA scaffolds to improve the osteoconduction of bone grafts. Future research is necessary to fully elucidate the potential use of PBLG-g-HA/PLLA scaffolds for bone tissue engineering or orthopedic surgery.
